# Risks of Infection with SARS-CoV-2 Due to Contaminated Surfaces: A Scoping Review

**DOI:** 10.3390/ijerph182111019

**Published:** 2021-10-20

**Authors:** Marjan Mohamadi, Awa Babington-Ashaye, Agnès Lefort, Antoine Flahault

**Affiliations:** 1 Faculty of Medicine, Institute of Global Health, University of Geneva, 1202 Geneva, Switzerland; Marjan.mohamadi@etu.unige.ch (M.M.); antoine.flahault@unige.ch (A.F.); 2 Service de Médecine Interne, Hôpital Beaujon, Clichy and IAME, UMR1137, INSERM and Université de Paris, 75006 Paris, France; Agnes.lefort@aphp.fr

**Keywords:** COVID-19, fomites, contaminated surfaces, transmission, SARS-CoV-2

## Abstract

The COVID-19 outbreak is a global health concern. Understanding the transmission modes of the SARS-CoV-2 virus is key to limit the spread of the pandemic. A lack of knowledge about the possibility of SARS-CoV-2 transmission and infection through contaminated surfaces is noticeable and recent studies have stated conflicting findings. This scoping review aims to understand the risks of contaminations via fomites better. Relevant publications were selected through Google Scholar, Web of Science, PubMed, Embase, Medline, and Cochrane Library, with related keywords. PRISMA-ScR guidelines were followed. Out of the 565 articles found, exclusion criteria were applied, duplicates removed, and a total of 25 articles were finally included in the study. The included documents were assessed by the contamination risk: “low” (37.5%), “high” (16.7%), “plausible” (8.3%), “unlikely” (8.3%) risk, and “insufficient evidence” (29.2%). Research in hospital settings was found as the main setting in the reviewed papers, which precisely indicated the risk of contaminated surfaces. This scoping review underscores the risk of SARS-CoV-2 infection via contaminated surfaces assessed as low in the majority of the reviewed articles. Further evaluation of the risk of the virus transmission by fomites and providing adequate information on its infectivity via contaminated surfaces in real-life conditions is essential.

## 1. Introduction

The coronavirus disease 2019 (COVID-19) outbreak is a global health concern [1], with an undoubtedly serious impact affecting people’s lives in various aspects, including healthcare, economic, and social factors [2,3]. The novel coronavirus was firstly detected in Wuhan in December 2019 [4]. On 12 March 2020, due to the high infectivity of SARS-CoV-2 [5] and numerous reported cases and deaths, the World Health Organization (WHO) declared the COVID-19 as a pandemic [6]. The virus SARS-CoV-2 belongs to the Coronaviridae family, some of whose viruses are associated with respiratory tract infections [7].

Understanding of the transmission modes of SARS-CoV-2 is a crucial matter. From a general perspective, the transmission of the respiratory virus has a few known routes, which are mainly (a) direct contact (person-to-person), (b) indirect contact by contaminated surfaces (fomites), and (c) airborne transmission by droplets or aerosols [8]. Based on the evidence to date, the transmission of SARS-CoV-2 is stated to be mostly airborne via droplets or aerosols through close contact with infected individuals [9,10]. However, other previous studies have suggested the possibility of hand to face transmission of pathogens by touching contaminated objects and surfaces, otherwise known as fomite transmission [11,12,13,14,15,16]. Moreover, face-touching behavior has been understood as a self-inoculation for microbial transmission [11,12,17,18]. In addition, some other studies underscore that contaminated surfaces could play a key role in the transmission of pathogens [13,14,15,16].

However, in the specific case of the SARS-CoV-2 virus, recent studies on the risk of fomites transmission have stated various findings. Most authors have demonstrated a low risk of contamination [11,19,20,21], while fewer authors suggested a higher risk [22,23].

For instance, as Kraay et al. stated, “while direct transmission is important, our model suggests that fomites can also transmit, which is important for exposures that are not in-person. Therefore, fomites transmission may be an important source of risk [22]”. Another study emphasized a key role of fomites transmission, as “the results showed that moderate protein concentration in droplets markedly increased the infectivity of SARS-CoV-2, suggesting that a protein-rich medium like airway secretions could protect the virus when it is expelled and may enhance its persistence and transmission by contaminated fomites. Accordingly, it is plausible that fomites infected with SARS-CoV-2 play a key role in the indirect transmission of COVID-19 [24].”

However, Goldman believes that “the chance of transmission through inanimate surfaces is very small and only in instances where an infected person coughs or sneezes on the surface, and someone else touches that surface soon after they cough or sneeze (within 1–2 h) [19]. Another study has found that “despite prolonged viability of SARS-CoV-2 under laboratory-controlled conditions, uncultivable viral contamination of inanimate surfaces might suggest low feasibility for indirect fomite transmission [25].” In addition, findings from Moore et al. demonstrated that “the concentration of viral RNA was low and ranged from <10 to 460 genomic copies/m3 air. Infectious virus was not recovered from any of the PCR-positive samples analyzed.” [26].

Therefore, in this present scoping review, we aim to provide a comprehensive overview of the literature about the risk of SARS-CoV-2 transmission via fomites in order to participate in a better understanding of relevant approaches to mitigate the propagation of the COVID-19 pandemic.

## 2. Materials and Methods

A scoping review of peer-reviewed and grey literature was conducted to identify the risks of infection with SARS-CoV-2 by contaminated surfaces.

A general literature search was first done on the SARS-CoV-2 virus to have a preliminary overview of the concepts and previous studies on this specific topic. Several studies on the SARS-CoV-2 virus were related to the risk of infection, such as contact and droplet transmission, airborne transmission, fomite transmission, and the possibility of transmission by animals. Importantly, this scoping review solely focused on resources related to the risks of infection with the SARS-CoV-2 due to contaminated surfaces. This review follows the framework of the Preferred Reporting Items for Systematic Review and Meta-analysis for Scoping Reviews (PRISMA-ScR) guideline.

### 2.1. Search Strategy

In order to broadly capture existing research on the defined topic, relevant publications were gathered using the search engines of Google Scholar, Web of Science, PubMed, Embase, Medline, and Cochrane Library.

Firstly, the search for the relevant literature was conducted using the following keywords in the titles of the articles: (SARS-CoV-2) OR (Novel Coronavirus) OR (COVID-19) AND (Fomites) OR (surface) OR (Inanimate surfaces) OR (Contaminated surfaces) OR (Environmental contamination) AND (Transmission) OR (Pathogen transmission) OR (Disease Transmission) AND (Viability) OR (Stability) OR (Survival) OR (Persistence).

**Figure 1:**
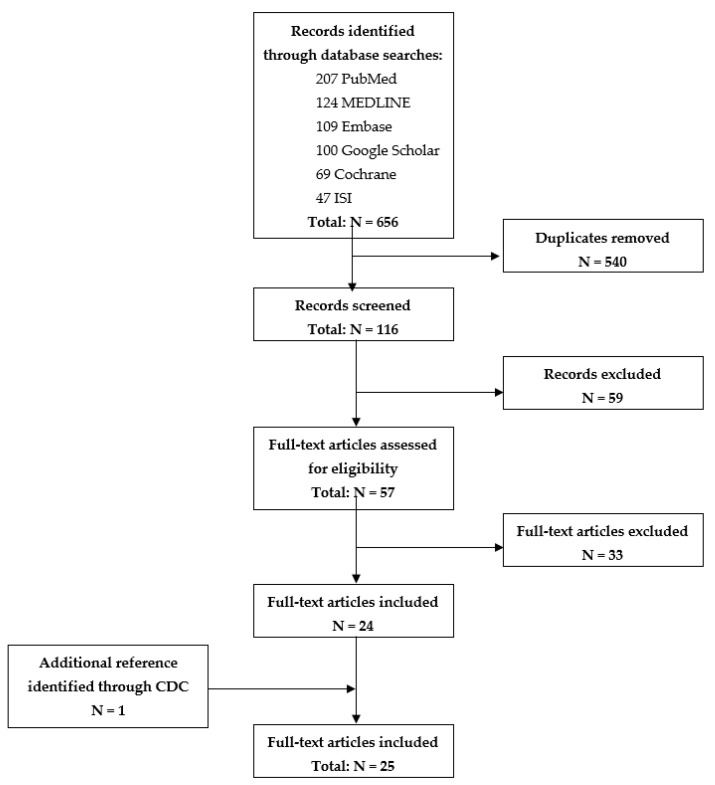
PRISMA flowchart.

The search was then broadened by including the ‘Abstracts’ in the search field. The methodology process used is summarized in Figure 1 (PRISMA flowchart).

Additionally, the search was also conducted by Mesh terms in PubMed as follows: (SARS-CoV-2) AND (Fomites) AND (Disease Transmission, Infectious) AND (Microbial viability) OR (Survival). The World Health Organization (WHO) and Centers for Disease Control and Prevention (CDC) websites were analyzed for relevant publications, which were considered as additional publications from other sources.

In order to find potential additional relevant sources, identification and screening of further articles from references were undertaken. After completing the search with all relevant publications, duplicates were omitted.

### 2.2. Inclusion and Exclusion Criteria

The inclusion criteria were: (i) English-language publications related to the potential risks of infection by contaminated surfaces with the SARS-CoV-2 virus; (ii) all types of scientific publications such as editorials, viewpoints, articles, guidelines, etc., were included; (iii) any relevant settings such as fomites in hospitals, public places or in-house, for instance.

Exclusion criteria included publications related to other findings on COVID-19 disease and its clinical aspects or other routes of transmission of the virus, for instance, as examples. In addition, publications that did not imply a correlation between variables, such as the viral stability or virus load with the risk of infection, for example, were excluded.

### 2.3. Data Extraction

The relevant dataset was extracted and imported into an Excel extraction table by the main researcher (M.M.) This table includes the title, author(s), date of publication, study location focus, type of publication, the main topic, key findings, contamination risk assessment, and limits. The other co-authors screened publications to ensure their relevance and eligibility. The selected articles were imported by EndNote X7. The process of selection is reported in Figure 1.

## 3. Results

The search performed in the different databases underscores various interesting findings related to the risk of infection with SARS-CoV-2 from contaminated surfaces. The key results can be summarized as follow:

From the 25 documents selected for this review, 24 were retrieved through the database searches and 1 from the website of the CDC.

As mentioned in Figure 1, of a total of 565 publications found in the databases and SARS-CoV-2 source-related websites, 116 publications remained after removing the duplicates and then were screened by titles and abstracts. At this stage, 59 of the reviewed articles were classified as out of the scope of our research question and, therefore, were excluded. The full text of the remaining 57 publications was assessed, and 24 articles finally met the inclusion criteria.

Importantly, some excluded articles did not exactly state the risk of infection by fomites but had investigated some features of the virus that might lead to infectivity, but the relationship between those features and infectivity was not precisely discussed.

In addition, several excluded articles discussed the stability of the virus in different environmental conditions, but a lack of evidence on the relationship between the stability features and the risk of infection by contaminated surfaces was observed. Contrastingly, 17 articles from this category discussed a potential correlation and were included in our scoping review. 

Five out of the 24 selected articles were surveyed in Europe, namely France, Switzerland, Germany, Italy, and England. Two of them were from the USA and one article, respectively, from Israel, China, and Singapore.

We could not identify a specific study setting in the remaining articles classified as Not Applicable, most of which were review articles and editorial letters. Articles since the beginning of the COVID-19 pandemic in early 2020 were assessed, and the last search was performed on 10 February 2021.

Amongst the included articles, ten out of 24 were review articles, which stated a clear conclusion about the risk of infection by fomites contaminated with SARS-CoV-2. Amongst the articles which have precisely indicated the risk of contaminated surfaces with SARS-CoV-2, five of them performed the research in hospital settings, three of them in community settings including schools, offices and high-touch surfaces in a city, one in a laboratory environment, and finally one in a quarantine household.

Appendix Table A1, Table A2 and Table A3 summarize the main features of the included publications.

Based on our review, the main topics of the relevant publications found were: (I) persistence of the SARS-CoV-2 on fomites or inanimate surfaces, (II) risk of transmission of SARS-CoV-2 from fomites, (III) infectivity of SARS-CoV-2 on surfaces, (IV) modes of transmission, (V) nosocomial transmission of SARS-CoV-2 from surface environments. 

Moreover, the articles were assessed by the contamination risk and were classified by “low”, “high”, “plausible”, and “unlikely” risk. Furthermore, a category was defined as “insufficient evidence”. Nine articles out of 24 (37.5%) assessed a low probability of transmission, and four articles out of 24 (16.7%) stated a high transmission probability.

**Figure 2:**
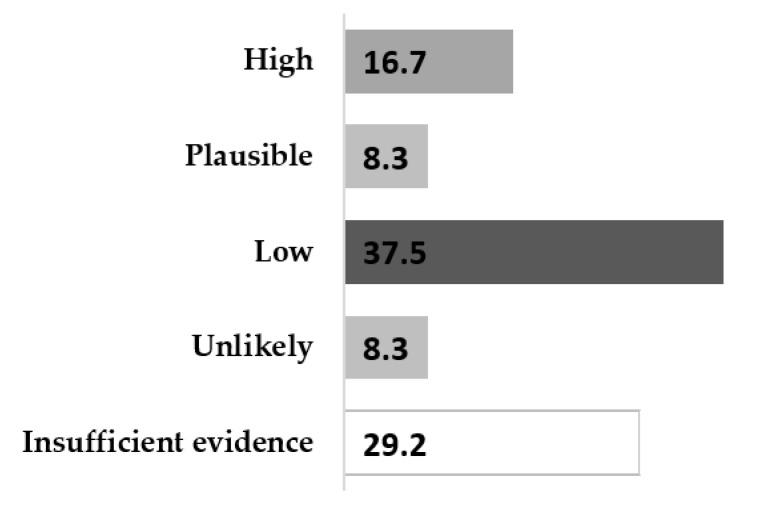
Contamination risk assessment of SARS-CoV-2 with contaminated surfaces (%).

Importantly, seven out of the 24 articles (29.2%) indicated not having enough evidence to determine the risk of SARS-CoV-2 infection by contaminated surfaces. These findings are summarized in Figure 2, and a detailed review of the risk assessment can be found in Table 1.

## 4. Discussion

This scoping review was performed to evaluate the available literature related to the risks of infection transmission via contaminated surfaces with SARS-CoV-2.

Our research highlights a noticeable variability in the findings of articles assessing the risk of transmission via fomites classified as follows: low or high possibility, unlikeliness, plausibility, or lack of adequate evidence to identify the risks of infection transmitted by fomites.

While previous studies on different microorganisms have demonstrated the existing risk of transmission by contaminated surfaces [13,14,15,16,17,18], surprisingly, only six out of 24 of the articles had indicated the “plausibility” or high risk of infection via fomites in the case of SARS-CoV-2 contamination.

We found that 29.2% of the reviewed articles stated an absence of enough evidence, and most of the articles, i.e., 45.8%, concluded a low probability or unlikeliness. Interestingly, those articles were mostly focused on the following conclusions: (i) the existence of the virus on surfaces does not prevail the risks of infection by the virus, and (ii) there is a lack of information about the infectious dose of SARS-CoV-2 on the surfaces to be transmitted in order to cause infection [32,35,38,39]. For instance, “The infectious dose of SARS-CoV-2, namely the average number of viral particles required to establish an infection for COVID-19 is unknown” as stated by Xue et al. 2020 [36]. Another rationale observed to support the low probability of transmission of the SARS-CoV-2 via contaminated surfaces is the demonstration that viruses cannot reproduce outside the host, and the observation of the rare occurrence of touching surfaces contaminated with viral loads high enough to be infective and the subsequently touch face membranes such as eyes, or nose [11,39].

In addition, as mentioned by the World Health Organization (WHO), “it is difficult to disentangle the relative contributions of inhaled droplets and contaminated surfaces because people who have come into contact with potentially infectious surfaces have generally also been in close contact with infected individuals”.

Moreover, there are differences between real-life and laboratory conditions, which lead to lower risks in the real living environment, especially when hygiene protocols and cleaning procedures are followed [11,29,33].

From another standpoint, the long persistence of the virus on surfaces might cause a high possibility of infection via contaminated surfaces [24,28]. In one reviewed study focused on hospital settings, with the assumption made that the concentration of the virus might be more important, fomites were identified as potential sources of the virus spread [25]. On the contrary, three other studies performed in the same settings concluded that the risk of transmission and infection is not high [21,26,31].

Interestingly although this scoping review aimed to identify specifically the risk of infection via fomites, we could observe debates about the duration of the virus presence on different surfaces and the environmental effects on it. For instance, “in laboratory-controlled conditions and at the ambient temperature, SARS-CoV-2 lost its infectivity completely by day 4” [31].

We also consider it crucial to mention that studies demonstrated that higher temperature, sunlight, and UV radiation highly lead to the inactivation of SARS-CoV-2 [23,37]. Those are important factors to be understood in the real-life context of the SARS-CoV-2 life cycle. In addition, studies demonstrated that the RNA of common viruses, such as SARS-CoV-2, Influenza, and MERS-CoV, can persist on surfaces for days after they have lost their infectivity [23], therefore, detecting viral RNA on surfaces cannot solely provide information about the infectivity of the virus [35]. Thus, such information has to be taken with the necessary scrutiny to avoid potential incomplete conclusions about the probability of getting infected via fomites in real-life conditions.

## 5. Conclusions

Given the date of the last search performed, articles published after 10 February 2021, were not integrated in our review. Some recent publications on that topic were found, confirming the relevance of this topic.

Conclusively, the main outcome of this scoping review is that the risk of SARS-CoV-2 infection via contaminated surfaces was assessed as low in the majority of the reviewed articles. Further evaluation of the risk of the virus transmission by fomites and adequate information on its infectivity via contaminated surfaces in real-life conditions are essential. Those investigations would participate in setting more efficient guidelines to limit the spread of the SARS-CoV-2. Until more evidence on the risk of the virus transmission by fomites in real-life situations can be gathered, it remains important to follow disinfection guidelines and, most importantly, respect physical distancing and the use of masks to limit the propagation of the COVID-19 pandemic. Finally, the authors acknowledge that scientific data and related issues regarding the SARS-CoV-2 virus and potential variants evolve rapidly, but at the time of the research, no sufficient data was available mentioning possible variants and fomites transmission. Therefore, it appears key to take into consideration those elements in future research.

## Figures and Tables

**Table 1:** Comments from the reviewed articles regarding the level of risk of SARS-CoV-2 infection through contact with contaminated surfaces.

**High**	“While direct transmission is important, our model suggests fomites can also transmit, which is important for exposures that are not in-person. Therefore, fomite transmission may be an important source of risk.” [22] “After reviewing ‘Similarities between SARS-CoV-2 and Other Coronaviruses’, ‘Effect of Media, Temperature, Relative Humidity, UV Irradiation, and Material-Type on SARS-CoV-2 Persistence’, the researchers concluded “the virus will persist on high-touch surfaces long enough to spread to new individuals.” [23] “Interpretation of findings for SARS-CoV-2 strongly suggest that the environment can serve as a medium of transmission of SARS-CoV-2, through touch contamination and subsequent self-inoculation of mucous membranes by a non-infected individual coming into contact with a contaminated environmental surface or fomite.” [27] “Our data showed that SARS-CoV-2 infectivity was remarkably preserved in the presence of proteins, regardless of the type of surface.”; “The results showed that moderate protein concentration in droplets markedly increased the infectivity of SARS-CoV-2, suggesting that a pro-tein-rich medium like airway secretions could protect the virus when it is expelled and may enhance its persistence and transmission by contaminated fomites. Accordingly, it is plausible that fomites infected with SARS-CoV-2 play a key role in the indirect transmission of COVID-19.” [24]
**Plausible**	“These findings suggest that the hospital environment could potentially be a source of virus spread, including among HCWs, patients, and visitors.” [25] “Our results indicate fomite transmission of SARS-CoV-2 is plausible since the virus can remain viable and infectious on surfaces up to days.” [28]
**Low**	“The work supports the current perception that contaminated surfaces are not a primary mode of transmission of SARS-CoV-2.”; “The risks posed by contact surfaces in 30 communities are low for community infection prevalence rates ranging from 0.2–5%.” [11] “The chance of transmission through inanimate surfaces is very small, and only in instances where an infected person coughs or sneezes on the surface, and someone else touches that surface soon after the cough or sneeze (within 1–2 h).” [19] “Findings suggest that environmental contamination leading to SARS-CoV-2 transmission is unlikely to occur in real-life conditions, provided that standard cleaning procedures and precautions are enforced.”; “transmission is unlikely to occur in real-life conditions, provided that standard cleaning procedures and precautions are enforced.” [29] “The risk of transmission via touching contaminated paper is low.” [20] “The results indicate that at that early time of SARS-CoV-2 outbreak research in Germany the contamination of the domestic environment is negligible during quarantine measured with the current state of the art methods. We could not detect any viral RNA in air samples and only 3.36% of all fomite samples.”; “This study supports the hypothesis that indirect environmental transmission may only play a minor role, which needs clarifications in further studies.” [21] “Toilet bowl and sink samples were positive, suggesting that viral shedding in stool could be a potential route of transmission.” [30] “The estimated risk of infection from touching a contaminated surface was low (less than five in 10,000) by quantitative microbial risk assessment, suggesting fomites play a minimal role in SARS-CoV-2 community transmission.”; “our results are consistent with fomite-mediated transmission of COVID-19 being possible but likely a secondary pathway.” [31] “Despite prolonged viability of SARS-CoV-2 under laboratory-controlled conditions, uncultivable viral contamination of inanimate surfaces might suggest low feasibility for indirect fomite transmission.”; “Aerosol or indirect transmission from inanimate surfaces around hospitalized or quarantined COVID-19 patients is not supported by the data presented in this study” [25]. “The concentration of viral RNA was low and ranged from <10 to 460 genomic copies/m3 air. Infectious virus was not recovered from any of the PCR-positive samples analyzed.” [26]
**Unlikely**	“Data on the transmissibility of coronaviruses from contaminated surfaces to hands were not found”; “The viral load of coronaviruses on inanimate surfaces is not known.” [32] “Our data suggest that although environmental contamination may occur in real-life conditions, it might be less extensive than hitherto recognized. Moreover, the inability of the SARS-CoV-2 RNA collected from the CPAP helmet to infect susceptible cell monolayers suggests that recent contamination of plastic surfaces, which apparently maintain SARS-CoV-2 infectivity for several hours, is unlikely to contribute to nosocomial spread.” [33]
**Insufficient evidence**	“Fomite transmission, i.e., viral dissemination via a material, including a door handgrip, door-bell, or inhalator, also has a critical contribution to the virus spread.”; “Survival duration of the COVID-19 causing virus on surfaces is not certainly known” [34] “Indirect transmission of COVID-19 has been assumed to be possible via fomites although direct evidence is currently not available.”; “The virus has been detected in hospital and household settings but detection of viral RNA on surfaces does not provide any information about viral infectivity or viability.” [35] “The infectious dose of SARS-CoV-2, namely the average number of viral particles required to establish an infection for COVID-19 is unknown.”; “It is currently unclear what role the surface chemistry plays in viral survival, infectivity, and denaturation, and the role of the local environment is unclear.” [36] “Fomite transmission would depend on the surface characteristics, which can affect virus survival and can help determine the extent of spread of the disease.” [37] “Overall, there was an inability to align SARS-CoV-2 contaminated surfaces with survivability data; and also a knowledge gap on fomite contribution to SARS-CoV-2 transmission.” [38] “Virus detection does not necessarily represent an infectious dose of SARS-CoV-2. Although SARS-CoV-2 may be transmitted via direct and indirect contact by touching contaminated sur-faces or medical equipment, followed by touching mouth, nose, or eyes, it remains unknown what portion of the transmission is attributable to a fomite.” [39] “The important ways of transmitting the virus are through Droplets, infected hands, and skin-to-skin contact, as well as inanimate surface contact.” [40]

## Appendix A

**Table A1:** Detailed information of review articles.

No	Reference	Publication Date	Study Location	Type of Record	Contamination Risk Assessment	Study Setting
1	Mondelli et al. [29]	2020 Sep	NA	Correspondence	Low	NA
2	Goldman [19]	2020 July	NA	Comment	Low	NA
3	Kampf [32]	2020 Feb	NA	Original article	Unlikely	NA
4	Hoseinzadeh et al. [34]	2020 July	NA	Review article	Not enough evidence	NA
5	Kampf et al. [35]	2020 Sep	NA	Review article	Not enough evidence	NA
6	Bueckert et al. [23]	2020 Nov	NA	Review article	High	NA
7	Xue et al. [36]	2020 Nov	NA	Review article	Not enough evidence	NA
8	Wathore et al. [37]	2020 Aug	NA	Review article	Not enough evidence	NA
9	Bedrosian et al. [38]	2020 Nov	NA	Review article	Not enough evidence	NA
10	Pitol and Julian [11]	2020 Nov	Switzerland	Original article (Pre-Print)	Low	Community setting
11	Kraay et al. [22]	2020 Aug	US	Original article (Pre-Print)	High	Schools Offices
12	Ong et al. [27]	2020 July	NA	Review article	High	NA
13	Kanamori [39]	2021 Jan	NA	Letter to editor	Not enough evidence	NA
14	Eslami and Jalili [40]	2020 May	NA	Review article	Not enough evidence	NA
15	Pastorino et al. [24]	2020 Sep	France	Research Letter	High	Laboratory setting
16	Van Doremalen et al. [28]	2020 April	US	Correspondence	Plausible	NA
17	Colaneri et al. [33]	2020 May	Italy	Original article	Unlikely	Hospital (emergency unit)
18	Ren et al. [20]	2020 April	NA	Review article	Low	NA
19	Döhla et al. [41]	2020 Jun	Germany	Original article (Pre-print)	Low	Quarantinehousehold
20	Ong et al. [21]	March 2020	Singapore	Original article	Low	Hospital
21	Harvey et al. [30]	2020 Dec	U.S.A	Original article	Low	Community setting
22	Ben-Shmuel [31]	2020 Dec	Israel	Original article	Low	Hospital: Isolation unit Quarantine hotel
23	Ye et al. [25]	April 2020	China (Wuhan)	Original article	Plausible	Hospital
24	Moore et al. [26]	2020 Nov	England	Original article	Low	Hospital

**Table A2:** Key findings of review articles.

No	Reference	Main Topic	Key Findings
1	[29]	Risk of transmission of SARS-CoV-2 from fomites	“Findings suggest that environmental contamination leading to SARS-CoV-2 transmission is unlikely to occur in real-life conditions, provided that standard cleaning procedures and precautions are enforced.”
2	[19]	Risk of transmission of SARS-CoV-2 from fomites	“The chance of transmission through inanimate surfaces is very small, and only in instances where an infected person coughs or sneezes on the surface, and someone else touches that surface soon after the cough or sneeze (within 1–2 h).”
3	[32]	Persistence of coronaviruses on inanimate surfaces	“Data on the transmissibility of coronaviruses from contaminated surfaces to hands were not found” “The viral load of coronaviruses on inanimate surfaces is not known.”
4	[34]	Modes of SARS-CoV-2 transmission	“Fomite transmission, i.e., viral dissemination via a material, including a door handgrip, door-bell, or inhalator, also has a critical contribution to the virus spread.” “Survival duration of the COVID-19 causing virus on surfaces is not certainly known”
5	[35]	Modes of SARS-CoV-2 transmission	“Indirect transmission of COVID-19 has been assumed to be possible via fomites although direct evidence is currently not available.” “The virus has been detected in hospital and household settings but detection of viral RNA on surfaces does not provide any information about viral infectivity or viability”
6	[23]	Infectivity of SARS-CoV-2 on surfaces	“After reviewing ‘Similarities between SARS-CoV-2 and Other Coronaviruses’, ‘Effect of Media, Temperature, Relative Humidity, UV Irradiation, and Material-Type on SARS-CoV-2 Persistence’, the researchers concluded “the virus will persist on high-touch surfaces long enough to spread to new individuals”
7	[36]	Infectivity of SARS-CoV-2 on surfaces	“The infectious dose of SARS-CoV-2, namely the average number of viral particles required to establish an infection for COVID-19 is unknown”. “It is currently unclear what role the surface chemistry plays in viral survival, infectivity, and denaturation, and the role of the local environment is unclear.”
8	[37]	Modes of SARS-CoV-2 transmission	“Fomite transmission would depend on the surface characteristics, which can affect virus survival and can help determine extent of spread of the disease.”
9	[38]	Risk of infection by fomites transmission	“Overall, there was an inability to align SARS-CoV-2 contaminated surfaces with survivability data; and also, a knowledge gap on fomite contribution to SARS-COV-2 transmission.”
10	[11]	Risk of infection by fomites transmission	“The work supports the current perception that contaminated surfaces are not a primary mode of transmission of SARS-CoV-2”. “The risks posed by contacting surfaces in 30 communities are low for community infection prevalence rates ranging from 0.2–5%.”
11	[22]	Risk of infection by fomites transmission	“While direct transmission is important, our model suggests fomites can also transmit, which is important for exposures that are not in-person. Therefore, fomite transmission may be an important source of risk”
12	[27]	Modes of the virus transmission	“Interpretation of findings for SARS-CoV-2 strongly suggest that the environment can serve as a medium of transmission of SARS-CoV-2, through touch contamination and subsequent self-inoculation of mucous membranes by a non-infected individual coming into contact with a contaminated environmental surface or fomite.”
13	[39]	Risk of infection by fomites transmission	“Virus detection does not necessarily represent an infectious dose of SARSCoV-2. Although SARS-CoV-2 may be transmitted via direct and indirect contact by touching contaminated surfaces or medical equipment, followed by touching mouth, nose, or eyes, it remains unknown what portion of transmission is attributable to a fomite.”
14	[40]	Modes of the virus transmission	“The important ways of transmitting the virus are through Droplets, infected hands, and skin-to-skin contact, as well as inanimate surface contact”
15	[24]	Infectivity of SARS-CoV-2 on surfaces	“Our data showed that SARS-CoV-2 infectivity was remarkably preserved in the presence of proteins, regardless of the type of surface.” “The results showed that a moderate protein concentration in droplets markedly increased the infectivity of SARS-CoV-2, suggesting that a protein-rich medium like airway secretions could protect the virus when it is expelled and may enhance its persistence and transmission by contaminated fomites. Accordingly, it is plausible that fomites infected with SARS-CoV-2 play a key role in the indirect transmission of COVID-19.
16	[28]	Infectivity of SARS-CoV-2 on surfaces	“Our results indicate fomite transmission of SARS-CoV-2 is plausible, since the virus can remain viable and infectious on surfaces up to days.”
17	[33]	Risk of infection by fomites transmission	“Our data suggest that although environmental contamination may occur in real-life conditions, it might be less extensive than hitherto recognized. Moreover, the inability of the SARS-CoV-2 RNA collected from the CPAP helmet to infect susceptible cell monolayers suggests that recent contamination of plastic surfaces, which apparently maintain SARS-CoV-2 infectivity for several hours, is unlikely to contribute to nosocomial spread.”
18	[20]	Risk of infection by fomites transmission	“The risk of transmission via touching contaminated paper is low.”
19	[41]	Risk of infection by fomites transmission	“The results indicate that at that early time of SARS-CoV-2 outbreak research in Germany the contamination of the domestic environment is negligible during quarantine measured with the current state of the art methods. We could not detect any viral RNA in air samples and only 3.36% of all fomite samples.” “This study supports the hypothesis that indirect environmental transmission may only play a minor role, which needs clarifications in further studies.”
20	[21]	Nosocomial transmission of SARS-CoV-2 from surface environmental	“Toilet bowl and sink samples were positive, suggesting that viral shedding in stool could be a potential route of transmission”
21	[30]	Risk of infection by fomites transmission	“The estimated risk of infection from touching a contaminated surface was low (less than 5 in 10,000) by quantitative microbial risk assessment, suggesting fomites play a minimal role in SARS-CoV-2 community transmission.” “our results are consistent with fomite-mediated transmission of COVID-19 being possible but likely a secondary pathway”
22	[31]	Infectivity of SARS-CoV-2 on surfaces	“Despite prolonged viability of SARS-CoV-2 under laboratory-controlled conditions, uncultivable viral contamination of inanimate surfaces might suggest low feasibility for indirect fomite transmission. “Aerosol or indirect transmission from inanimate surfaces around hospitalized or quarantined COVID-19 patients is not supported by the data presented in this study.”
23	[25]	Contamination assessment of and by surfaces in hospital settings	“These findings suggest that the hospital environment could potentially be a source of virus spread, including among HCWs, patients, and visitors”
24	[26]	Nosocomial transmission of SARS-CoV-2 from surface environmental	“The concentration of viral RNA was low and ranged from <10 to 460 genomic copies/m3 air. Infectious virus was not recovered from any of the PCR-positive samples analyzed.”

**Table A3:** Recommendations and limitations of review articles.

No	Reference	Main Topic	Gaps/Limitations/Recommendations for Further Studies
1	[29]	Risk of transmission of SARS-CoV-2 from fomites	Not Provided
2	[19]	Risk of transmission of SARS-CoV-2 from fomites	“None of these studies (which have been checked by the author) present scenarios akin to real-life situations.”
3	[32]	Persistence of coronaviruses on inanimate surfaces	Not Provided
4	[34]	Modes of SARS-CoV-2 transmission	Not Provided
5	[35]	Modes of SARS-CoV-2 transmission	“It has to be mentioned that in most studies only PCR was per-formed for RNA. But detection of viral RNA on surfaces does not provide any information about viral infectivity or viability”
6	[23]	Infectivity of SARS-CoV-2 on surfaces	“Each experiment had a specific lower limit of detection (LOD) at which infectious virus could not be discerned, and distinct sampling points which influenced data. Consequently, the recorded durations of viability are inexact and presented as a range in this review. Studies often failed to report the LOD and/or choice of sampling points, so the applicability of their data is questionable. There are no available data on the transmissibility of coronaviruses from inoculated surfaces to hands.”
7	[36]	Infectivity of SARS-CoV-2 on surfaces	“A systematic study on the lifetime of infectious viruses on a range of existing polymers under ambient conditions would be useful for those choosing which personal protective equipment to use. The theoretical basis for describing viral particle interactions at synthetic surfaces is not well developed.”
8	[37]	Modes of SARS-CoV-2 transmission	“Nature of the role played by Particulate Matter should be thoroughly examined so as to understand its efficacy in aiding or reducing the transmission and survival of these viruses. The lack of information on particulate matter effect prevents complete understanding on the stability of coronavirus on particulate matter and PM-contaminated common surfaces. it is high time that the effect of confounding factors such as composition of particulate matter, should be explored in detail to understand the chances of real-life survivability and transmissibility of viruses such as SARS-CoV-2.”
9	[38]	Risk of infection by fomites transmission	“Pre-prints were included in review, bias was not assessed beyond noting preprint percentage, and after completing the review we (based on information available) determined to focus the review on surface contamination, stability, and disinfection. Also, no data was found in this review that suggests the actual likelihood of contracting COVID-19 via fomites; in subsequent systematic reviews, it is recommended to include patient case studies to assess potential fomite base transmission across settings. We do not feel these limitations impact “ The model is based on data of SARS-CoV and Murine hepatitis virus (MHV-1) infection in mice by intranasal administration. Extrapolating the model from mice to people and from MHV-1 and SARS-CoV to SARS-CoV-2, introduces uncertainty in infection risk estimates, but we did not consider this here. An additional limitation is that the dose-response relationship was determined using virus as measured in units of Plaque Forming Units (PFU) and therefore a ratio of genome copies to PFU is needed. Model parameters used for virus transfer and decay rates are determined experimentally in laboratory conditions and could be different in environmental conditions. Also, prevalence rates modeled here are assumed to correspond directly with the percent of people who are infected and contact the surface with a hand contaminated by coughing. In reality, an unknown fraction of infected people would likely either: 1) stay at home, or 2) not cough directly on their hand.” the results presented herein.”
10	[11]	Risk of infection by fomites transmission	“The model is based on data of SARS-CoV and Murine hepatitis virus (MHV-1) infection in mice by intranasal administration. Extrapolating the model from mice to people and from MHV-1 and SARS-CoV to SARS-CoV-2, introduces uncertainty in infection risk estimates, but we did not consider this here. An additional limitation is that the dose-response relationship was determined using virus as measured in units of Plaque Forming Units (PFU) and therefore a ratio of genome copies to PFU is needed. Model parameters used for virus transfer and decay rates are determined experimentally in laboratory conditions and could be different in environmental conditions. Also, prevalence rates modeled here are assumed to correspond directly with the percent of people who are infected and contact the surface with a hand contaminated by coughing. In reality, an unknown fraction of infected people would likely either: 1) stay at home, or 2) not cough directly on their hand.”
11	[22]	Risk of infection by fomites transmission	Not provided
12	[27]	Modes of the virus transmission	“Further studies to clarify the extent and relative importance of each of these transmission routes, as well as the patient, disease and environmental factors that affect each medium, are urgently needed to allow policymakers to risk-stratify and tailor infection control recommendations.”
13	[29]	Risk of infection by fomites transmission	“Indicating the necessity of further studies on survival and contamination as well as clinical evidence on fomite transmission”
14	[40]	Modes of the virus transmission	Not provided
15	[24]	Infectivity of SARS-CoV-2 on surfaces	Not provided
16	[28]	Infectivity of SARS-CoV-2 on surfaces	Not provided
17	[33]	Risk of infection by fomites transmission	“The timing of swabbing, which was relatively close to the cleaning procedures and the effectiveness of flocked swabbing for environmental sampling. Sample collection from the environment was set-up on the basis of a precise workflow and it cannot apply to surfaces that are not systematically cleaned, e.g., computer keyboards, telephones, multi-parameter monitors, infusion pumps, etc. to cite but a few.”
18	[20]	Risk of infection by fomites transmission	Not provided
19	[41]	Risk of infection by fomites transmission	“It should be noted that these data were generated under laboratory conditions. It can be assumed that households in quarantine have a cleaning regime, but even under these conditions viral RNA could be found on fomites in the households. A further characterization of different cleaning systems (frequency, cleaning agents, ventilation of rooms, etc.) would be necessary; however, this effect could only be validly estimated in observational studies, since a large bias towards social desirability can be 280 expected in surveys “
20	[21]	Nosocomial transmission of SARS-CoV-2 from surface environmental	“This study has several limitations. First, viral culture was not done to demonstrate viability. Second, due to operational limitations during an outbreak, methodology was inconsistent and sample size was small. Further studies are required to confirm these preliminary results.”
21	[30]	Risk of infection by fomites transmission	“Uncertainty in key QMRA model parameters could lead to an overestimate of the risk.” the ratio of RNA to viable virus in clinical samples may be different than this ratio in environmental surface samples. We did not attempt to culture live virus from any of our surface samples and therefore cannot determine the viability or infectivity of the SARS-CoV-2 detected in our samples. Future work is needed to confirm the relationship between SARS-CoV-2 RNA concentrations and viable virus on surfaces and to determine if infective SARS-CoV-2 can be recovered from fomites in community settings.”
22	[31]	Infectivity of SARS-CoV-2 on surfaces	“There was a delay between onset of symptoms and the actual sampling in patients’ rooms. Therefore, at the time of sampling, these patients might not have shed viable virus, as suggested by studies that showed culturable viruses in respiratory samples up to the 8th or 9th day of illness. For that reason, we have noted new patients with recent disease onset in Hospital A and the quarantine hotel and sampled around them. The CPE assay has a 10 pfu/mL limit of detection that is comparable to a CT value of 34, therefore a very low level of viability cannot be ruled out. Unforeseen technical issues could have compromised viability of the virus after sampling. We addressed this limitation by collecting nearly 100 samples in three separate campaigns and maintaining strict cold storage conditions after sampling and during transport. Moreover, re-culturing of all the negative cultures was performed to overcome any problems of culture adaptation of freshly isolated virus.”
23	[25]	Contamination assessment of and by surfaces in hospital settings	“SARS-CoV-2 culture samples were not collected; thus, exact bioburden levels were unable to be determined. No air samples were collected during the investigation. This study focused on hospital surface contamination of SARS-CoV-2 RNAs as a surrogate of exposure to SARS-CoV-2. We were limited in our ability to characterize other exposure factors, such as other exposure routes, frequencies, and duration. A lack of resources also meant that we were unable to conduct a comprehensive exposure study for the HCWs working in the hospital in the midst of the ongoing outbreak.”
24	[26]	Nosocomial transmission of SARS-CoV-2 from surface environmental	“When sampling the healthcare environment, many variables can impact the results obtained. This can make interpretation of the data difficult, particularly if a frame of reference is lacking. In this study and to provide context, agar contact plates were used to provide an aerobic bacterial colony count and an indication of surface cleanliness.”
